# Diet and Lifestyle in Nonalcoholic Fatty Liver Disease

**DOI:** 10.1155/2020/3495082

**Published:** 2020-12-15

**Authors:** Roberto Martínez-Beamonte, Sergio Acín, Adela Ramírez-Torres, María Ángeles Navarro

**Affiliations:** ^1^CIBER de Fisiopatología de la Obesidad y Nutrición, Instituto de Salud Carlos III, Madrid, Spain; ^2^Instituto Agroalimentario de Aragón, CITA-Universidad de Zaragoza, Zaragoza, Spain; ^3^Molecular Genetics Group, Department of Physiology and Biochemistry, School of Medicine, Universidad de Antioquia, Medellin, Colombia; ^4^Cedars Sinai, Los Angeles, USA; ^5^Departamento de Bioquímica y Biología Molecular y Celular, Facultad de Veterinaria, Universidad de Zaragoza, Zaragoza, Spain

The liver is an essential metabolic organ which governs body energy metabolism connected with adipose tissue and skeletal muscle among other tissues. The prevalence of obesity has reached epidemic proportions in many countries around the world and continues to grow every year which is caused by multiple factors, with diet and lifestyle being the most researched and therefore most important.

Nonalcoholic fatty liver disease (NAFLD) is one of the several metabolic complications associated with obesity. The pathology of NAFLD is difficult to recognize or diagnose especially in early stages without a biopsy and therefore can remain undetected for significant time allowing the disease to progress.

The diagnosis of NAFLD is crucial to be able to start adequate treatment including changes in diet and lifestyle in the first stage of the disease when the pathology is reversible and prevent the development of severe forms of the disease such as nonalcoholic steatohepatitis (NASH) or the irreversible cirrhosis stage.

When the liver becomes damaged, this can lead to some metabolic alterations that have a severe and multifaceted impact in type 2 diabetes mellitus (T2DM), visceral obesity, and cardiovascular disease related to elevated plasmatic cholesterol, triglycerides, transaminases, and others that indicate hepatic disorders and oxidative stress.

This special issue aims to bring articles to assemble the latest progress to combat NAFLD, NASH, and cirrhosis mainly related to overweight and obesity which are increasingly prevalent in today's society. The manuscripts received by the journal were carefully selected pursuing this objective.

I. Grgurevic et al.'s review paper “Natural History of Nonalcoholic Fatty Liver Disease: Implications for Clinical Practice and an Individualized Approach” summarizes the current knowledge on the natural history of NAFLD and suggests there is not still strong evidence of significant association between the inflammatory component of NAFLD and the adverse clinical outcomes. The authors propose older age (>50 years) and presence of T2DM and some genetic variants as indicators of increased risk of having liver fibrosis.

The review made by Z. Miao et al. showed new insights and basis for medical therapy on adipose metabolic diseases, related to diet, and more concrete with the consumption of vitamin D. This review summarized the relationship between vitamin D and the adipose tissue that represent the major storage of vitamin D and classified its biological roles and functionalities with the regulation and development on adipogenesis and other metabolic diseases, calcium homeostasis, and energy metabolism.

H. Ou et al. have written a cross-sectional study with 225 patients with NAFLD, smokers, and nonsmokers, to evaluate a lifestyle parameter over NAFLD. Liver-significant fibrosis was diagnosed by liver stiffness using a FibroScan. The results indicate that the risk of fibrosis in smokers with NAFLD is significantly higher, showing higher changes in significant liver fibrosis and advanced liver fibrosis in these patients, with influence of age and AST levels too.

T. Himoto et al. made a cross-sectional study with diet intervention during 6 months in patients with NAFLD to determine the nutritional and dietary factors associated with muscle volume loss in these patients with presarcopenia, sarcopenia, and sarcopenic obesity. The authors indicate that there are different factors that regulate muscle volume loss in male and female NAFLD patients.

Another interesting paper written by B. Martínez et al. used a special diet during 2 months in a porcine animal model to induce fatty liver. After the dietary fatty liver induction, the authors used 10 mg/kg/day of melatonin to reverse the fatty liver, feeding the animals with the same steatosic special diet. The results showed that the melatonin group does not increase steatosis, with a decrease of MDA levels.

A. M. Akinnuga et al. presented the first work using bredemolic acid in a diet-induced prediabetic rat model during 12 weeks. The prediabetes model showed an increase in AST and ALT, liver glycogen and triglycerides, and lipid peroxidation and a decrease in SREBP1 expression. The bredemolic acid administration decreased liver enzyme concentration and hepatic oxidative stress and improved antioxidant enzymes such as SOD and GPx, thus improving liver function.

